# Tumor-Associated Macrophages Correlate With Prognosis in Medulloblastoma

**DOI:** 10.3389/fonc.2022.893132

**Published:** 2022-07-04

**Authors:** Jin Zhang, Xia Yuan, Yuan Wang, Jingjing Liu, Zhigang Li, Shuting Li, Yan Liu, Xiaojun Gong, Yanling Sun, Wanshui Wu, Liming Sun, Shuxu Du, Tianyou Wang

**Affiliations:** ^1^Department of Pediatrics, Beijing Shijitan Hospital, Capital Medical University, Beijing, China; ^2^Hematology Center, Beijing Key Laboratory of Pediatric Hematology Oncology, National Key Discipline of Pediatrics (Capital Medical University), Key Laboratory of Major Disease in Children, Ministry of Education, Beijing Children’s Hospital, Capital Medical University, National Center for Children’s Health, Beijing, China; ^3^State Key Laboratory of Natural and Biomimetic Drugs, School of Pharmaceutical Sciences, Peking University, Beijing, China; ^4^Hematologic Disease Laboratory, Hematology Center, Beijing Key Laboratory of Pediatric Hematology Oncology, National Key Discipline of Pediatrics (Capital Medical University), Key Laboratory of Major Disease in Children, Ministry of Education, Beijing Pediatric Research Institute, Beijing Children’s Hospital, Capital Medical University, National Center for Children’s Health, Beijing, China

**Keywords:** medulloblastoma, macrophage, phenotype, prognosis, immunotherapy

## Abstract

**Purpose:**

Macrophage polarization plays an essential role in the tumor microenvironment of brain tumors. However, the role of tumor-associated macrophages (TAMs) in medulloblastoma still remains controversial. Thus, we investigated the distribution of macrophages in medulloblastoma tissues and analyzed the association of TAM recruitment and medulloblastoma patients’ outcomes.

**Methods:**

We obtained a total of 71 paraffin sections from patients with medulloblastoma, and detected the activated phenotype (M1/M2) by monoclonal antibodies for CD68, HLA-DR and CD163 with multiple fluorescence immunohistochemistry method. The number of polarized macrophages was quantified using the InForm software. Outcomes were analyzed according to clinical data and quantified macrophage data.

**Results:**

The study revealed that TAMs were significantly higher in sonic hedgehog (SHH) medulloblastoma than in other subgroups, and M1 macrophages in metastatic group were significantly higher than those in non-metastatic group. A Kaplan-Meier survival analysis and multivariate Cox regression model showed the correlation of high percentage of total macrophages (*P* = 0.038, HR = 0.241) and M1 macrophages (*P* = 0.034, HR = 0.333) with good 5-year progression-free survival (PFS); however, M2 macrophages had no correlation with survival of medulloblastoma patients (*P*> 0.05).

**Conclusion:**

High percentage of total macrophages and M1 macrophages are correlated with good outcome of medulloblastoma patients. TAMs might be a target of therapy.

## Introduction

Medulloblastoma is the most common primary pediatric malignancy of the central nervous system, which is divided into wingless (WNT), sonic hedgehog (SHH) and non-WNT/non-SHH molecular subgroups (1). Multimodal treatment consisting of surgery, radiation therapy and chemotherapy has significantly improved its long-term survival (2). However, approximately 30% of medulloblastoma patients present with metastases at diagnosis and their survival has remained unfavorable (3, 4).

Tumorigenesis is a complex and dynamic process. In recent years, the role played by the tumor microenvironment in promoting or inhibiting brain tumor growth has caused more concerns (5, 6). Tumor-associated macrophages (TAMs) are a key component of the tumor microenvironment that can either support tumor growth by promoting angiogenesis and immune suppression or suppressing tumor growth via pro-inflammatory effect (7). Studies have suggested that TAMs are the major immune cells in brain tumor microenvironment; moreover, macrophage polarization plays an essential role in the growth and progression of brain tumors (8–10).

At present, the role of TAMs in prognosis of medulloblastoma is still controversial. Margol et al. (11) first reported that TAMs was significantly higher in SHH medulloblastomas compared to other subgroups. Other study also showed that TAMs played an active role in SHH medulloblastoma by inhibiting tumor growth (12). A reduction or repolarization of TAMs can result in accelerated tumor progression. However, a recent study revealed that effective reduction of TAMs had a negligible role in medulloblastoma recurrence and metastatic spread (13). On the other hand, Lee et al. (14) suggested that high percentage of M1 rather than M2 showed worse outcome in SHH medulloblastoma patients, contrary to the common view on role of M1 phenotype in prognosis.

In our study, we described the characteristics of the activated phenotype (M1/M2) of TAMs in medulloblastoma and investigated the correlation between TAM recruitment and prognosis.

## Materials and Methods

### Patients and Samples

With the approval of the Institutional Review Board, tissue samples were collected from 71 medulloblastoma patients who underwent surgical resection between 2015 and 2020. All the patients were newly diagnosed and treatment naive at the time of surgery and received the same treatments after surgery. Maximal surgical resection was followed by risk-adapted cranio­spinal irradiation and adjuvant chemotherapy (15). Treatment strategies were performed on the German Society of Pediatric Oncology and Hematology (GPOH) Protocol HIT 2000 (16–18). Patient data were obtained by medical record review including age, gender, date of surgical operation, pathological and molecular subtype, tumor stage and treatment outcome. Informed consent was obtained from all of the patients and/or their parents.

### Immunofluorescence Staining and Antibodies

Multiplex immunofluorescence staining was performed on 5-μm-thick fixed tissue sections. Sequential staining steps were as follows. Slides were deparaffinized in xylene, rehydrated through graded alcohols and then underwent microwave treatment in EDTA antigen repair solution for antigen retrieval. They were blocked and stained with commercially available anti-CD68 (1:300; 0030300020; Panovue Inc.), anti-CD163 (1:200; 0026200025; Panovue Inc.) and anti-HLA-DR (1:300; 0036500020; Panovue Inc.) at the room temperature for 1 h. Next, incubation with HRP-labeled secondary antibody (0013001006, Panovue Inc.) was performed at the room temperature for 10 min. Each antigen was labeled by distinct Opal fluorophores. Tyramide signal amplification (TSA) technique was used to amplify the immunofluorescence signal. Microwave treatment was performed to remove the antibody-TSA complex after every staining cycle. Multiplex antibody panel was optimized as follows: CD68, Opal 520; CD163, Opal 570; HLA-DR, Opal 650. Nuclei were subsequently stained with DAPI (1:100) and the slides were dried and sealed with anti-fluorescence quencher. Finally, the stained tissue slices were observed under fluorescence microscope.

### Image Acquisition and Digital Image Analysis

Each whole-tissue section was scanned for multispectral imaging on Vectra-Polaris Automated Quantitative Pathology Imaging System (Akoya Biosciences). Five fields (image size: 930 μm×700 μm) were selected randomly from hot spot areas in the digital image (×20) per slice. The number of fluorescent signal-positive cells and the proportion of the total cell number were performed with the inForm image analysis software (Akoya Biosciences) along the programed cell-count algorithm. Then, the mean percentage of positive cells in five fields was calculated.

### Statistical Analysis

SPSS 24.0 software (IBM, Armonk, NY, USA) was used for statistical analysis. The variables disobeying normal distribution were presented as the median (range). The Mann-Whitney test was used to determine differences between numerical variables not obeying normal distribution. Univariate survival analyses were performed using Kaplan-Meier method, and differences in survival between two groups were compared with the log-rank test. Multivariate survival analyses were performed using the Cox regression model, and the odds ratio (OR) and 95% confidence interval (CI) were calculated. A P value <0.05 was considered to be statistically significant. Progression-free survival (PFS) was defined as the time from the date of surgery to date of progression. Overall survival (OS) was defined as the time from the date of surgery to date of death or last follow-up. The last follow-up time was December 31th, 2021.

## Results

### Patient Characteristics

**Figure 1:**
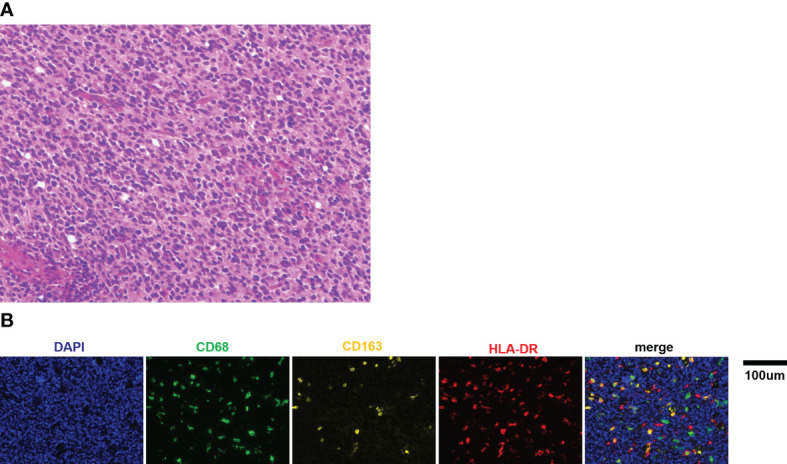
**(A)** Representative picture of H&E staining for medulloblastoma (×400). **(B)** Representative immunofluorescence images of CD68, CD163, and HLA-DR staining in medulloblastoma tissue sections. Scale bar, 100μm.

The clinical characteristics of all patients are shown in **Table 1**. A total of 71 patients (54 boys, 17 girls) were enrolled in the study. All patients were pathologically diagnosed with medulloblastoma (**Figure 1A**). The median age was 7.1 years (range 0.8-18 years). Eight cases were under 3 years old, and 63 cases were 3 years of age or older. The patients were all with known pathological types (43 CMB, 23 DNMB, 2 MBEN, 3 LCA) and molecular subgroups (3 WNT, 29 SHH, 39 non-WNT/non-SHH). Twenty-five cases presented with metastasis at diagnosis, while 46 cases were without metastasis.

**Table 1:** Clinical characteristics of medulloblastoma patients.

Characteristics	Number of patients (%)
Age (year)	
<3	8 (11)
≥3	63 (89)
Gender	
boy	54 (76)
girl	17 (24)
Pathological type	
CMB	43 (61)
DNMB	23 (32)
MBEN	2 (3)
LCA	3 (4)
Molecular subtype	
WNT	3 (4)
SHH non-WNT/non-SHH	29 (41) 39 (55)
Metastasis	
Yes	25 (35)
No	46 (65)

CMB, classic medulloblastoma; DNMB, desmoplastic/nodular medulloblastoma; MBEN, medulloblastoma with extensive nodularity; LCA, large-cell/anaplastic medulloblastoma; WNT, wingless; SHH, sonic hedgehog.

### Activated Macrophage Recruitment in the Medulloblastoma Patients

It has been reported that there is a pronounced activation of TAMs in medulloblastoma tissues: the percentage of TAMs was significantly increased in medulloblastoma (9.8% ± 0.3%) compared to normal cerebellum (1.2% ± 0.04%) (12). Thus, this study focused on the TAMs of medulloblastoma tissue in this study. Multiplex immunofluorescence and image analysis were conducted to identify macrophage recruitment. The representative immunofluorescence images are shown in **Figure 1B**. The recruited proportion of CD68, CD163, and HLA-DR positive macrophages were quantified based on the set threshold. CD68, CD163 and HLA-DR are well-established markers for TAMs M1/M2 phenotypes (19–22). CD68^+^ cells represented total macrophages (M_total_), and CD68^+^ HLA-DR^+^ CD163^−^cells were defined as M1 macrophages, CD68^+^ CD163^+^ HLA-DR^−^ cells were defined as M2 macrophages. The results showed that the median proportion of M_total_ was 5.47% (range: 1.72% - 17.39%), M1 was 1.33% (range: 0.26% - 4.33%) and M2 was 0.47% (range: 0.02% - 1.88%). In addition, we found large amounts of CD68^+^ CD163^+^ HLA-DR^+^ cells (named as M_mix_ for convenience), with median proportion of 2.13% (range: 0.28% - 7.53%).

### Correlations of TAM Recruitment With Common Clinical Characteristics

**Figure 2:**
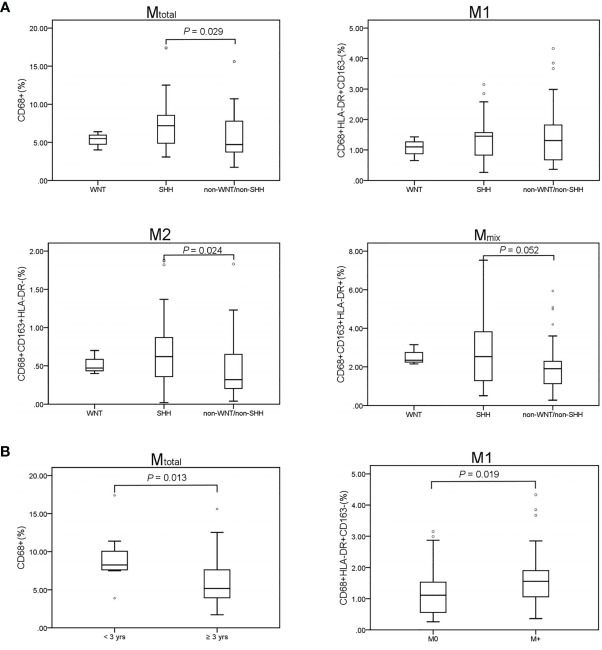
TAM recruitment in medulloblastoma patients. Results of the Mann–Whitney U test are presented. Only significant results are displayed. **(A)** Comparison of TAM recruitment in the medulloblastoma subgroups. WNT (n = 3), SHH (n = 29), non-WNT/non-SHH (n = 39). M_total_ (*P* = 0.029) and M2 (*P* = 0.024) were significantly higher in the SHH subgroup than those in the non-WNT/non-SHH subgroup. M_mix_ percentage tended to be higher in SHH than that in non-WNT/non-SHH (*P* = 0.052). No difference was revealed in M1 proportion (*P* = 0.941). **(B)** M_total_ was significantly higher in children younger than 3 years (*P* = 0.013). M1 in metastatic group was significantly higher than that in non-metastatic group (*P* = 0.019). M0, non-metastatic group; M+, metastatic group. °, outlier.

We compared the percentage of different types of TAMs among different medulloblastoma subgroups (**Figure 2**). There were no significant differences in the proportions of TAMs in WNT subgroup compared with SHH and non-WNT/non-SHH groups (*P* > 0.05), which may be due to the small number of patients in WNT subgroup. M_total_ (*P* = 0.029) and M2 (*P* = 0.024) macrophages were both significantly higher in SHH than those in non-WNT/non-SHH. The percentage of M_mix_ tended to be higher in SHH than that in non-WNT/non-SHH (*P* = 0.052). There was no difference in M1 percentage between these two subgroups (*P* = 0.941). There were similar results between SHH and the other two groups including WNT and non-WNT/non-SHH (M_total_, *P* = 0.024; M2, *P* = 0.025; M_mix_, *P* = 0.066; M1, *P* = 0.820). These results showed that SHH group had more infiltration of macrophages.

The proportion of M_total_ was higher in patients under 3 years old (*P* = 0.013). Then, the children were further divided into three age groups: 0-3, 4-10, and 11-18 years old. M_total_ was higher in both 0-3-year-old and 11-18 year-old children than that of 4-10 -year-old children (*P* = 0.004 and 0.046, respectively). M1 was higher in 11-18 -year-old children than that of 4-10-year-old children (*P* = 0.013). There were no differences in all types of TAMs between 0-3 and 11-18 years old groups (*P* > 0.05). M1 macrophages in patients with metastasis were significantly higher than that in non-metastatic patients (*P* = 0.019). There was no difference in TAM proportions among pathological types and sex (*P* > 0.05).

### Association Between TAM Recruitment and Patient Survival

**Figure 3:**
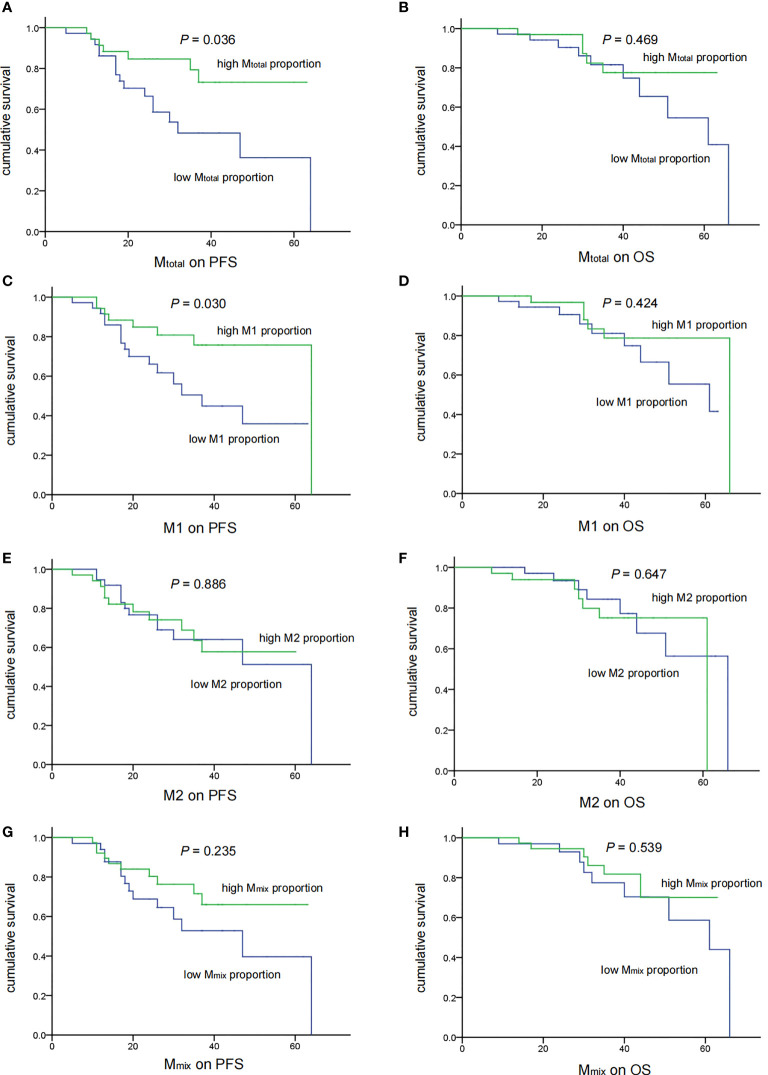
Associations of TAM recruitment with treatment outcomes in medulloblastoma patients (n = 71). There were significant correlations of low M_total_ **(A)** (*P* = 0.036) and M1 **(C)** (*P* = 0.030) recruitment with shorter PFS. No significant correlation of M_total_ **(B)** and M1 **(D)** with OS was observed (*P* = 0.469 and 0.424 respectively). M2 and M_mix_ recruitment showed no correlation with the PFS and OS **(E–H)**.

Statistical analysis was conducted to demonstrate the correlation between TAM recruitment and patient survival. All patients were divided into two groups, the low- and high-percentage groups, according to the median of the macrophage proportion. The PFS and OS analyses were performed using the Kaplan-Meier analysis and the log-rank test (**Figure 3**). Five-year PFS was significantly poorer in low M_total_(*P* = 0.036) and M1 (*P* = 0.030) groups than in high groups, however, there was no difference in 5-year OS between low and high M_total_ or M1 groups (*P* > 0.05). Moreover, M2 and M_mix_ proportion had no significant effect on 5-year PFS and OS of these patients (*P*> 0.05).

Then, we analyzed the relationship between clinical features and prognosis of medulloblastoma patients (**Table 2**). In univariate analysis, high M_total_ and M1 were associated with better 5-year PFS (*P* = 0.036 and 0.030, respectively). Metastasis was associated with poor 5-year OS (*P* = 0.031). Next, the common prognostic factors were introduced into multivariate Cox regression analysis together. The results showed that high M_total_ (*P* = 0.038, HR = 0.241, 95% CI = 0.063 - 0.927) and M1 (*P* = 0.034, HR = 0.333, 95% CI = 0.120 - 0.920) macrophages were independently correlated with better 5-year PFS (**Table 2**). As expected, metastasis was independently correlated with a shorter 5-year PFS (*P* = 0.012, HR = 3.235, 95% CI = 1.293 - 8.095, **Table 2**) and 5-year OS (*P* = 0.014, HR = 4.987, 95% CI = 1.383 - 17.987, **Table 2**), while M2 and M_mix_ proportion, age and sex had no significant influence on 5-year PFS and OS (*P* > 0.05, **Table 2**).

**Table 2:** Univariate and multivariate analyses of clinical variables associated with PFS and OS.

Clinical Factor	PFS				OS			
	Univariate	Multivariate			Univariate	Multivariate		
	*P* value	*P* value	HR	95% CI	*P* value	*P* value	HR	95% CI
M_total_	0.036	0.038	0.241	0.063 - 0.927	0.469	0.548	0.593	0.108 - 3.265
M1	0.030	0.034	0.333	0.120 - 0.920	0.424	0.358	0.548	0.152 - 1.978
M2	0.886	0.090	2.412	0.871 - 6.677	0.647	0.209	2.295	0.628 - 8.387
M_mix_	0.235	0.663	1.258	0.448 - 3.534	0.539	0.999	1.000	0.260 - 3.852
Age	0.076	0.102	1.100	0.981 - 1.232	0.263	0.700	0.967	0.815 - 1.147
Sex	0.354	0.262	0.526	0.171 - 1.617	0.808	0.390	0.523	0.119 - 2.291
Metastasis	0.147	0.012	3.235	1.293 - 8.095	0.031	0.014	4.987	1.383 - 17.987

HR, hazard ratio; CI, confidence interval.

## Discussion

At present, the potential role of immune microenvironment in promoting or inhibiting human medulloblastoma progression has not been fully clarified (23). In this study, we found that TAMs increased specifically in the SHH subgroup. We also investigated the correlation between TAM recruitment and treatment outcome. It was showed that high M_total_ and M1 macrophages predicted better PFS, however, TAMs made no significant effect on OS.

The SHH subgroup is common and the most investigated type in medulloblastoma, approximately accounting for 30% of all patients (15, 24). The SHH signaling pathway plays an important role in actively orchestrating many aspects of cerebellar development and maturation (25). SHH subgroup is characterized by constitutive activation of the SHH signaling pathway leading to abnormal proliferation of the cerebellar cells (26). Our study showed that the quantity of TAMs was abundant in SHH medulloblastoma, which was consistent with the reports in other literatures (10, 11, 14). Moreover, the proportion of M2 macrophages increase in SHH medulloblastoma. These may be due to the significant increase of monocyte chemotactic protein-1 (MCP-1/CCL2), which could recruit TAMs and promoting M2 macrophage polarization, in human SHH medulloblastoma (12, 27). Furthermore, it was reported that tumor-derived SHH directly acts on TAMs to promote M2 polarization, mediated by the transcription factor Krüppel-like factor 4 (Klf4) (28). In addition, other signaling pathways are also involved, such as signal transducer and activator of transcription 3 (STAT3), which is necessary to maintain SHH signaling and can enhance macrophage proliferation and survival through multiple pathways (29, 30). Augmented M1 recruitment in SHH subgroup was not observed. The underlying mechanism is unclear and needs to be explored. Meanwhile, the finding of higher percentage of macrophages in patients younger than 3-year-old in this study may be explained by the fact that SHH cases were mainly under 3 years old.

In this study, high M_total_ and M1 macrophages were associated with better short-term prognosis, which was consistent with the classical anti-tumor effect of M1 macrophages in various tumor types (31–33). However, there are controversial results in other studies. No significant association between survival and macrophage infiltration in any subgroups was found in one study (10), whereas a negative correlation between M1 and prognosis of SHH subgroup patients was observed in another (14). Furthermore, macrophage abrogation decreased the survival of murine models (12). It has been showed that genetic aberrations and stages of disease make different effect on immune microenvironments including macrophages (34, 35). For instance, TAMs are mainly composed of different proportions of both microglia and monocyte-derived macrophages, and the extent and composition of TAMs are different in molecular subtypes (36). It was noteworthy that there were many differences in the proportion of molecular subtypes, age and number of patients with metastatic disease between Lee et al’s and our study (14). Moreover, Lee et al. mainly investigated the correlation between TAM recruitment and prognosis in SHH subgroup, while we validated it in all the groups of patients. Thus, these contradictory findings may be related to the heterogeneity of patients with different proportion of molecular subtypes in different studies including ours. It also implies the complexity and the lack of understanding of macrophages. With regard to the noncorrelation of TAM counts with OS, TAMs at diagnosis may not affect the overall outcome as the composition of TAMs would change with treatment and disease progression (13, 36). Our data represented just the TAMs characterization before surgery and chemoradiotherapy. Dynamic observations on TAMs during treatment may contribute to prognosis evaluation more helpfully. Further research is needed while cerebrospinal fluid might be a potential alternative (37).

It is reported that the immunoreactivity pattern is age-related in medulloblastoma (38). In order to investigate whether and how patient age affects the TAMs profile, the patients were divided into three age groups. We found that 0-3-year-old and 11-18-year-old children had higher M_total_ than 4-10-year-old children, and 11-18-year-old children had higher M1 than 4-10-year-old children. It implies that differentially targeting TAMs is crucial in different age groups of patients, and we need to focus on 4-10-year-old patients who may have a poorer immunotherapeutic efficacy on TAMs due to the low macrophage count (39).

It is well-known that the patients with metastasis had a poor prognosis. In this study, however, we found that M1 macrophages increased significantly in patients with metastatic disease. Probably, M1 macrophages were highly recruited by some tumor suppressors to enhance the phagocytesis ability (40), although this mechanism could not eventually resist the invasion of the tumor. The exact underlying mechanisms remain to be clarified. There was relatively high proportion of patients with metastatic disease, therefore PFS correlation with M1 might be confounded by the number of metastatic samples in this case. However, Cox proportional hazard regression model showed that M1 and metastasis were both independently prognostic factors of PFS, which avoided the confounding factors.

In this study, there were high percentages of CD68^+^ CD163^+^ HLA-DR^+^ cells with a mixed signature of M1/M2. The markers of M1 and M2 can be co-expressed on the same cell. A high amount of mixed M1/M2-like polarized macrophages exists in a variety of tumors (41, 42). The value of these macrophages for prognostic evaluation is not clear. We did not find any correlation between M_mix_ and prognosis in this study. A recent research showed that tumor reduction was associated with the percentage of macrophages with mixed M1/M2 phenotypes, and the outcome of these therapeutic strategies targeting TAMs was influenced by the half-life of various macrophage phenotypes (43). The abundance of these macrophages indicates the plasticity of TAMs and the potential of targeting TAMs for immunotherapy in medulloblastoma. It also reflects that the paradigm of categorizing TAMs into M1 and M2 does not fully represent the phenotypic diversity and comprehensive functions of TAMs, especially in brain immunobiology (44, 45). TAMs with current widely accepted biomarkers could play a role in both pro-and anti-tumor functions (46). For example, TAMs expressing M2-associated markers may display M1-like functions under a wide variety of stimuli (47). However, one limitation of the present study is lacking of functional interrogation of TAMs. Further studies are necessary for a more in-depth investigation of their functions.

TAMs are a promising target for tumor immunotherapy. Existing therapeutic strategies targeting TAMs mainly include adjusting the TAM population, reverting TAMs into M1 macrophages, regulating macrophagic phagocytosis signals and engineering macrophages to trigger phagocytosis (48). M1 and M2 macrophages have high degree of plasticity and can be converted into each other upon tumor microenvironment changes or therapeutic interventions (49–51). Therefore, the attempts to convert TAMs into M1 macrophages or reduce M2 macrophages have potential value in treatment of medulloblastoma. At present, animal trials have showed an anti-tumor effect of STAT3 inhibitors and CSF1R inhibitors in medulloblastoma (30, 52, 53). Thus, this therapy strategy would probably improve the patient prognosis in the future.

## Conclusion

Higher total macrophages and M1 macrophages recruitment predicted a good outcome in medulloblastoma patients. TAMs might be a target of therapy for medulloblastoma.

## Data Availability Statement

The original contributions presented in the study are included in the article/supplementary material. Further inquiries can be directed to the corresponding authors.

## Ethics Statement

The studies involving human participants were reviewed and approved by Ethics Committee of Beijing Shijitan Hospital, Capital Medical University. Written informed consent to participate in this study was provided by the participants’ legal guardian/next of kin.

## Author Contributions

TW, SD, WW, and LS. designed experiments; JZ. carried out experiments; YW. assisted with multiplex immunofluorescence staining; XY and JZ. conducted image analysis; JZ, JL, YS, XG, SL, and YL. contributed to the acquisition of data and statistical analysis; JZ. wrote the manuscript; ZL and SD. revised the text of the manuscript. All authors contributed to the article and approved the submitted version.

## Funding

This work was supported by National Science and Technology Key Projects (2017ZX09304029001). Capital Health Development Scientific Research Special Project (2020-2-2047) and Fund of Beijing Shijitan Hospital of Capital Medical University(2021-C04).

## Conflict of Interest

The authors declare that the research was conducted in the absence of any commercial or financial relationships that could be construed as a potential conflict of interest.

## Publisher’s Note

All claims expressed in this article are solely those of the authors and do not necessarily represent those of their affiliated organizations, or those of the publisher, the editors and the reviewers. Any product that may be evaluated in this article, or claim that may be made by its manufacturer, is not guaranteed or endorsed by the publisher.

## Abbreviations

TME, Tumor microenvironment; TAM,Tumor-associated macrophage; SHH, Sonic hedgehog; WNT, Wingless; PFS, Progression-free survival; OS, Overall survival; GPOH, German Society of Pediatric Oncology and Hematology; TSA, Tyramide signal amplification; OR, Odds ratio; CI, Confidence interval; MCP-1, Monocyte chemotactic protein-1 ; STAT3, Signal transducer and activator of transcription 3.
